# Risks and Benefits of Using Social Media in Dermatology: Cross-sectional Questionnaire Study

**DOI:** 10.2196/24737

**Published:** 2021-02-24

**Authors:** Moshe Y Bressler, Eugene Grudnikoff, Yaakov Bressler, Rebecca Tamez, John G Zampella

**Affiliations:** 1 New York Institute of Technology College of Osteopathic Medicine Old Westbury, NY United States; 2 Department of Dermatology Jamaica Hospital Medical Center Richmond Hill, NY United States; 3 Department of Psychiatry South Oaks Hospital Amityville, NY United States; 4 Ronald O Perelman Department of Dermatology New York University New York, NY United States

**Keywords:** social media, dermatologist, generational differences, Instagram, Facebook, information quality, patient education, online content, risk, benefit, dermatology, cross-sectional, survey, online health information

## Abstract

**Background:**

Dermatological information on social media is often presented by nondermatologists. Increasing the online engagement of trained dermatologists may improve information quality, patient education, and care.

**Objective:**

Our study assesses dermatologists’ perceptions of social media and patterns of use to identify barriers limiting engagement.

**Methods:**

In our cohort study, a 36-item online survey was distributed to dermatologists in the United States; responses were captured on a 1-100 sliding scale.

**Results:**

Of 166 initiated surveys, 128 valid responses were submitted. Dermatologists showed greater concern for social media risk-related issues (mean 77.9, SD 15.1) than potential benefits (mean 61.8, SD 16.4; *P*<.001). Leading concerns were poor patient care, nonevidence-based information, and breaching patient privacy. Benefits included interphysician collaboration, patient education, and public health awareness. The most avid and enthusiastic social media users were millennials (mean total optimism score 67.5, SD 14.9) and baby boomers (mean total optimism score 63.1, SD 11.2) compared with Generation X dermatologists (mean total optimism score 52.2, SD 16.3, *P*<.001). Of 128 dermatologists, 103 (82.4%) plan on increasing their social media use (*P*=.003). Predictors showing an intent to increase future social media use were younger age, integration into professional use, and an optimistic view (r2=.39; *P*<.001).

**Conclusions:**

Dermatologists perceive the risk of social media to be considerable but still intend to increase its use, likely recognizing the value and importance of social media to the field.

## Introduction

Americans spend an average of 142 minutes per day on social media, and this number is expected to rise [1]. It is no surprise that patients have turned to social media for information regarding health care, with reports of more than 125 million Americans using social media to search for health-related information [2]. The use of social media as a health care resource has documented benefits. Studies point to improved patient well-being and empowerment through the use of social media, especially among those with new medical diagnoses [3].

Patient use of social media also has its downfalls, with little quality control or regulation of the information posted to social media platforms. Patients often encounter misinformation with potentially harmful outcomes [4,5]. Campaigns such as the #VerifyHealthcare movement encourages physicians on social media to validate their credentials to help identify posts with reliable medical information; however, the extent to which such interventions alter health literacy has not been evaluated [6].

Indeed, false information tends to spread 6 times faster on social media than factual information [7,8], and re-educating patients to correct false information can be challenging [9]. For health-related content, a physician’s engagement on social media is effective in spreading quality information and has the potential to reach millions of people [10]. The barriers preventing practitioners and experts from participating are likely multifactorial but may involve concerns over privacy violations [11], fear of litigation [12], and uncertainty surrounding patient-physician boundaries on social media [13].

Dermatologists were early adopters of social media, and many continue to make educational and relevant content for consumers. A recent study showed that “top influencer” dermatologists have large social media audiences and provide a valuable educational service to patients [14]. However, these influencers may not be sufficient to combat the gaps in public health education, as other studies show that as little as 4%-5% of dermatology-related content on Instagram is posted by board-certified dermatologists [2,15].

To increase dermatologist engagement and the positive outcomes associated with a strong physician presence on social media, we aim to understand the perceptions and behaviors of dermatologists in the United States using social media. With this understanding, we can appropriately guide policies to promote safe and effective participation on social media while mitigating risks.

## Methods

### Study Design

Using the SurveyMonkey tool (SVMK Inc), we created and distributed an anonymous, open, online survey of 36 questions (Multimedia Appendix 1) to board-certified dermatologists and dermatology residents enrolled in an Accreditation Council for Graduate Medical Education (ACGME)–accredited program in accordance with the Checklist for Reporting Results of Internet eSurveys (CHERRIES) [16]. Participants disclosing non-US–based practices were screened. Multiple entries were prevented by limiting duplicate IP address entries. The survey design was created using prior models [17] and piloted by 6 dermatologists and 2 dermatology residents.

The survey was distributed using an academic listserv (Association of Professors in Dermatology [APD]), which was distributed to 486 members and the private Facebook group “Board Certified Dermatologists,” with more than 4500 board-certified dermatologists. Survey questions used a 0-100 sliding scale, where 100 represented maximal agreement. Information collected included demographic data, social media usage patterns and preferences, and perceptions of social media, including positive and negative effects of social media and its effect on relationships. Upon completion, users were able to share the survey with their peers using our web landing page.

### Data Analysis

Data were stratified for credentials, degree, employment type, years of experience, years on social media, geographical region, favorite social media platform, and generational differences. We compared millennials (ages 23-38 years in 2019), Generation X (ages 39-54 years), and baby boomers (ages 55-73 years) using definitions outlined by Pew Research [18]. Chi-square and ANOVA tests were used for the analysis of categorical and continuous variables, respectively. When an omnibus ANOVA F-test revealed significant differences between multiple groups, we performed group-to-group post hoc analyses; the Fisher exact test was used due to low *n* in some cells. A *t* test was used for group comparisons with unequal variances. Variables associated at *P*<.1 with a response of “yes” or “maybe” regarding the intention to increase social media use were entered in a backward elimination multiple linear logistic regression model to identify variables independently associated with intent to increase social media use. All analyses were 2-sided with alpha set at .05, and they were conducted using JMP statistical software (version 9.0; SAS Institute Inc).

We created a scoring system to evaluate positive and negative perceptions of social media by calculating the net sum-average of all the responses in each category. Potential benefits yielded a total optimism score, risks and concerns yielded a total pessimism score (wherein a higher value indicates greater pessimism), and a positive or negative effect on relationships generated the total relationship scores (wherein a higher value indicates a positive effect on relationships).

﻿

## Results

### Respondent Demographics

Of 166 initiated surveys, 128 were valid—38 entries were disqualified from the analysis due to a location outside of the United States, or a nonphysician or nondermatologist status.

Of the 128 valid entries, 48 (37%) respondents were male and 80 (63%) were female, with an average age of 38.7 (SD 9.7) years and an average time in clinical practice of 9.3 (SD 9.2) years. Of the 128 respondents, 36 were residents (28%) and 93 (72%) were board-certified dermatologists; 117 (91%) had Doctor of Medicine (MD) degrees and 11 (9%) had Doctor of Osteopathic Medicine (DO) degrees; 71 (57%) were millennials, 42 (34%) were Generation X, and 12 (10%) were baby boomers. The respondents were evenly distributed by sex (*P*=.72) and geographical region (*P*=.34). Our sample was representative of the US dermatological workforce. Additional demographic, experience, and employment characteristics are reported in Table 1.

**Table 1:** Demographic characteristics of survey responders (n=128).

Variable		Total (n=128)	Millennial (n=71, 56.8%)	Generation X (n=42, 33.6%)	Baby boomer (n=12, 9.6%)	*P* value
Age in years, mean (SD)		38.7 (9.7)	31.9 (3.0)	44.1 (4.4)	59.6 (6.6)	<.001^a^
**Gender, n (%)**						.72
	Female	80 (62.5)	47 (66.2)	26 (61.9)	6 (54.6)	
	Male	48 (37.5)	24 (33.8)	16 (38.1)	5 (45.5)	
**Region, n (%)**						.34
	Midwest	17 (13.3)	9 (12.7)	6 (14.3)	2 (16.7)	
	Northeast	54 (42.2)	34 (47.9)	14 (33.3)	3 (25.0)	
	South	40 (31.3)	20 (28.2)	16 (38.1)	3 (25.0)	
	West	18 (14.1)	8 (11.3)	6 (14.3)	4 (33.3)	
Clinical experience in years, mean (SD)		9.3 (9.2)	3.5 (3.3)	7.3 (10.1)	29.4 (9.9)	<.001^b^
**Employment, n (%)**						.016^c^
	Academic institution	79 (61.7)	40 (58.0)	28 (66.7)	8 (66.7)	
	Equity owner of a group practice	4 (3.1)	0 (0.0)	3 (7.1)	1 (8.3)	
	Owner of a solo practice	12 (9.4)	5 (7.3)	4 (9.5)	3 (25.0)	
	Group practice, hospital or health care system	32 (25.0)	24 (34.8)	7 (16.7)	0 (0.0)	

^a^Age: all post hoc comparisons significant at *P*<.001.

^b^Years of clinical experience: all post hoc comparisons significant at *P*<.001.

^c^Employment: millennials are less likely to be employed as equity owners of a group practice vs. nonmillennials (*P*=.035) and more likely to be employed at a group practice or hospital (*P*=.007; baby boomers are less likely to be employed at a group practice or hospital (*P*=.036).

### Social Media Practices

Among the 128 respondents, 120 (93.8%) reported using social media across a variety of platforms, including Facebook (109/128, 85.2%), Instagram (85/128, 66%), and LinkedIn (51/128, 40%; Table 2), for an average of 45.9 (SD 35.2) minutes/day. Millennials had used social media for an average of 11.8 (SD 3.0) years, significantly longer than either GenX (mean 9.4, SD 3.6 years) or baby boomers (mean 6.2, SD 3.4 years; *P*<.001). The overall time spent on social media for professional use was 16.9 (SD 24.3) minutes/day and 31.1 (SD 22.0) minutes/day for personal use; millennials spent more total time on social media compared to baby boomers and GenX respondents. Unexpectedly, baby boomers’ time spent on social media for professional use (mean 17.3, SD 18.9 minutes/day) was comparable to that of millennials (mean 21.6, SD 28.6 minutes/day; *P*=.67).

Owners of private practices and solo practitioners spent more time on social media for professional use compared to all other respondents (mean 41.7, SD 41.5 minutes/day vs. mean 13.9, SD 20.2 minutes/day; *P*<.001), while physicians working at academic institutions spent less time on social media (mean 11.6, SD 18.6 minutes/day vs. mean 24.8, SD 29.9 minutes/day; *P*=.003). Overall, 44% (53/128) of physicians found Instagram to be the most valuable platform, followed by Facebook (49/128, 40.7%), and preferences varied by generation. Social media usage patterns and preferences are reported in Table 2.

**Table 2:** Social media patterns and preferences observed from the survey responses (n=128).

Variable		Total (n=128)	Millennial (n=71, 56.8%)	Generation X (n=42, 33.6%)	Baby boomer (n=12, 9.6%)	*P* value
Years of social media use, mean (SD)		10.6 (3.7)	11.8 (3.0)	9.4 (3.6)	6.2 (3.4)	<.001^a^
Personal time spent on social media, min/day, mean (SD)		31.1(22.0)	38.5(20.2)	22.7(21.9)	15.0 (12.8)	<.001^b^
Professional time spent on social media, min/day, mean (SD)		16.9 (24.3)	21.6 (28.6)	8.5 (13.1)	17.3 (18.9)	.021^b^
Total time spent on social media, min/day, mean (SD)		45.9 (35.2)	59.6 (34.7)	30.5 (29.2)	29.6 (25.5)	<.001^c^
Plan to increase social media use, n (%)		103 (82.4)	67 (94.4)	28 (66.7)	8 (66.7)	.001^d^
**Platforms with an active account, n (%)**						
	Facebook	109 (85.2)	62 (87.3)	36 (85.7)	8 (66.7)	.18
	Instagram	85 (66.4)	58 (81.7)	21 (50.0)	4 (33.3)	<.001^e^
	LinkedIn	51 (39.8)	25 (35.2)	19 (45.2)	5 (41.7)	.56
	Reddit	16 (12.5)	8 (11.3)	5 (11.9)	2 (16.7)	.87
	Snapchat	33 (25.8)	30 (42.3)	2 (4.8)	0 (0.0)	<.001^f^
	Twitter	29 (22.7)	14 (19.7)	11 (26.2)	3 (25.0)	.71
	WhatsApp	49 (38.3)	32 (45.1)	15 (35.7)	2 (16.7)	.15
	YouTube	35 (27.3)	18 (25.4)	12 (28.6)	3 (25.0)	.93
Most valuable platform, n (%)		Instagram, 53 (44.4)	Instagram, 42 (60.0)	Facebook, 22 (59.5)	Facebook, 6 (50.0)	.004^g^
**Location where social media is accessed, n (%)**						
	Home	114 (89.1)	66 (93.0)	35 (83.3)	10 (83.3)	.24
	Work	54 (42.2)	38 (53.5)	11 (26.2)	4 (7.6)	.014^h^
	During commute	32 (25.0)	24 (33.8)	5 (11.9)	2 (16.7)	.027^i^

^a^Years on social media: all post hoc comparisons significant at *P*<.033.

^b^Time spent on social media: all post hoc comparisons significant at *P*<.031.

^c^Time on social media above average: millennial>GenX, *P*<.001; millennial>baby boomer, *P*<.001.

^d^Plan to increase social media use: millennial>GenX, *P*<.001; millennial>baby boomer, *P*=.013.

^e^Active Instagram account: all post hoc comparisons significant at *P*<.02.

^f^Active Snapchat account: all post hoc comparisons significant at *P*<.036.

^g^Most valuable platform: Facebook, GenX>millennials, *P*<.001; Instagram, millennial>GenX, *P*<.001 , millennial>baby boomers, *P*<.001.

^h^Social media use at work: GenX>millennials, *P*<.001.

^i^Social media use during commute: millennial>GenX, *P*=0.014.

### Perceptions

**Figure 1:**
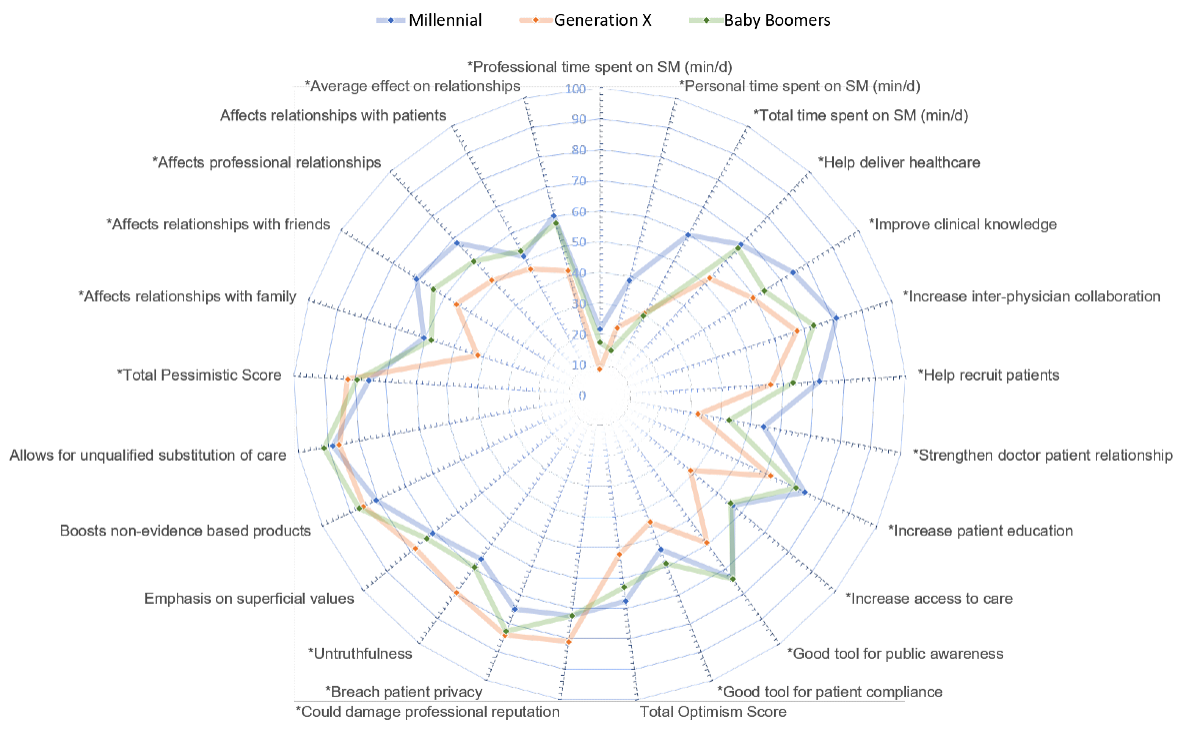
Generational differences of social media use and perceptions. Survey questions were answered on a 0-100 agreement scale. The radar chart demonstrates the average for each question grouped by generation. Questions calculated toward the total optimism score are highlighted teal, pessimistic questions are highlighted pink, and questions affecting relationships are highlighted yellow. (*) denotes statistically significant different answers by generation using a Fisher test, P≤.05. min/d: minutes per day; SM: social media.

Overall, dermatologists perceived that social media has many benefits and uses (total optimism score 61.8, SD 16.4). There was strong agreement that social media use increases patient education (69.4, SD 20.6), while less agreement concerning access to care or strengthening the doctor-patient relationship (50.3, SD 21.8, and 46.1, SD 24.7, respectively). Millennials (67.5, SD 14.9) and baby boomers (63.1, SD 11.2) were more optimistic about the benefits of social media than the GenX physicians (52.2, SD 16.3; *P*<.001 and *P*=.030, respectively). Attitudes and perceptions regarding social media are reported in Table 3 and Figure 1.

Dermatologists showed greater concern for risk-related issues on social media compared to potential benefits (mean score 77.9, SD 15.1 vs. mean score 61.8, SD 16.4, paired *t* test; *P*<.001). The greatest concerns were that social media use contributes to the substitution of proper dermatological care with unqualified providers (88.5, SD18.0), promotion of nonevidence-based products (82.1, SD 20.6), and the threat of breaching patients’ privacy (78.9, SD 19.9); however, this varied by generation, with millennials being less pessimistic than GenX dermatologists (*P*=.018).

Most dermatologists believed that social media use improves relationships with friends (65.3, SD 21.0) and professional colleagues (61.4, SD 22.3) but were more neutral about social media’s effect on relationships with patients (50.3, SD 21.2).

**Table 3:** Future users versus nonusers of social media (n=128).

Survey question		Response score by generation, mean (SD)									*P* value^a,b^
		Total (n=128)		Millennial (n=71, 56.8%)		Generation X (n=42, 33.6%)		Baby boomer (n=12, 9.6%)			
**Perceived benefits related to social media**											
	Help deliver health care	61.9 (24.2)	67.3 (24.7)		52.3 (22.3)		65.8 (19.5)		.005		
	Improve clinical knowledge	68.2 (24.6)	74.7 (20.5)		59.2 (27.9)		63.6 (22.9)		.003		
	Increase interphysician collaboration	75.3 (21.2)	81.3 (19.0)		67.6 (23.4)		73.3 (17.3)		.005		
	Help recruit patients	65.7 (22.1)	71.9 (21.2)		55.9 (23.3)		63.1 (11.8)		<.001		
	Strengthen doctor-patient relationship	46.1 (24.7)	54.4 (23.9)		32.7 (23.2)		43.0 (16.1)		<.001		
	Increase patient education	69.4 (20.6)	74.0 (22.6)		61.6 (16.4)		70.8 (15.6)		.008		
	Increase access to care	50.3 (21.8)	56.5 (19.5)		38.5 (22.4)		55.3 (20.6)		<.001^b^		
	Good tool for public awareness	68.8 (22.6)	73.8 (20.7)		59.5 (24.9)		73.9 (18.4)		.004^b^		
	Good tool for patient compliance	51.5 (22.2)	54.1 (23.1)		44.5 (21.0)		59.0 (17.9)		.040^b^		
	Total optimism score	61.8 (16.4)	67.5 (14.9)		52.2 (16.3)		63.1 (11.2)		<.001^b^		
**Perceived risks related to social media**											
	Could damage professional reputation	74.9 (21.4)	72.5 (23.3)		80.9 (15.8)		72.4 (22.5)		.11		
	Breach patient privacy	78.9 (19.9)	75.0 (21.4)		84.1 (16.3)		82.8 (17.1)		.044		
	Untruthfulness	70.1 (26.3)	65.9 (25.8)		79.5 (25.1)		69.4 (19.9)		.024		
	Emphasis on superficial values	72.9 (24.0)	70.6 (25.0)		78.2 (22.1)		73.3 (24.0)		.27		
	Boosts nonevidence-based products	82.1 (20.6)	80.5 (20.2)		85.2 (21.9)		86.6 (12.4)		.38		
	Allows for unqualified substitution of care	88.5 (18.0)	88.7 (18.0)		86.5 (20.1)		91.7 (9.3)		.66		
	Total pessimism score	77.9 (15.1)	75.5 (14.8)		82.4 (15.0)		79.4 (12.7)		.058		
**Perceived social media effect on relationships**											
	Affects relationships with family	54.1 (22.5)	60.3 (20.7)		41.9 (22.7)		57.8 (19.9)		.002^b^		
	Affects relationships with friends	65.4 (21.1)	70.7 (20.3)		55.4 (20.8)		64.4 (18.5)		.006		
	Affects professional relationships	61.3 (22.3)	68.0 (21.5)		51.4 (23.2)		59.9 (15.9)		.011		
	Affects relationships with patients	50.2 (21.2)	51.6 (26.0)		47.0 (18.5)		53.6 (7.9)		.68		
	Average effect on relationships	60.2 (19.1)	60.3 (20.7)		41.9 (22.7)		57.8 (19.9)		.002^b^		

^a^All significant *P* values ≤.05 for millennials vs. GenX.

^b^*P*≤.05 for GenX vs. baby boomer; there were no significant differences between millennials and baby boomers.

### Future Users vs. Nonusers

Of the 128 dermatologists surveyed, 103 (82.4%) are actively or considering increasing their social media usage. The variables independently associated with a plan to use more social media in the future were younger age (*P*=.023), use at work (*P*=.028), and average optimism (*P*<.001) in the logistic regression model using backward elimination. In contrast, other variables (eg, average pessimism, employment type, favorite social media platform) were codependent or not significant (final model r^2^=.390, *P*<.001).

## ﻿Discussion

### Principal Findings

Our survey of US dermatologists demonstrates that there are many perceived risks and benefits of social media. These views vary significantly across generations, yet our data suggest the perceived benefits outweigh the associated risks. Our study provides insight into physicians’ perceptions of social media; the results may serve as a guide to promoting dermatologist engagement on social media.

The total pessimism score revealed a general negativity surrounding social media use among dermatologists. Pessimism was driven by perceived risks of poor patient care, misinformation, damage to professional reputation, and privacy breaches, consistent with prior reports of social media risks [2,11–13] (Figure 1). Mitigating these risks will be essential for increasing the engagement of dermatologists on social media. Indeed, the risks associated with physicians sharing information online have been identified as a key area for social media research [13], with little data currently reported. Patient privacy violations resulting from posting and sharing patient photographs are common among some specialties [19] and represent real concern, as images can be downloaded and reshared, increasing patient exposure to privacy breaches. The lack of clear guidelines for sharing photos of patients online may cause physicians to avoid creating patient-centered content altogether [20].

Alternatively, there is promising optimism for social media use among dermatologists. The total optimism score calculated from our survey reflects the perceived benefits of social media, which include increased health care access, improved education, and improved public health (Figure 1). Despite a more prominent, uniform, overall pessimism score acknowledging inherent risks, a preponderance of those surveyed (103/128, 82.4%) plan to increase social media usage. Using a linear regression model, we found that optimism predicts an increase in future social media usage, while the pessimism score has no predictive value. This implies that perceived benefits outweigh the risks.

Additional predictors of increasing social media use are younger age and use at work. Dermatologists may be moving to social media for economic reasons, and younger physicians may consider a social media presence necessary to compete in a modern medical marketplace. This is likely a self-reinforcing process where physicians that use social media to recruit more patients will benefit most and feel more positively about it. Alternatively, physicians who are currently not using social media are not exposed to its benefits and therefore view social media as nonadvantageous. Studies suggest that 32% of people have made health decisions using social media [21] and may explain that one of the perceived benefits of social media discovered in our survey was the ability to recruit patients. A recent study by Murphy et al [22] found that 43% of all patients consider social media to be moderately to extremely important in choosing a dermatologist, particularly for patients seeking cosmetic procedures [23-25].

Perception of benefits and risks of social media varied by generation. Unexpectedly, millennials (ages 23-38 years in 2019) and baby boomers (ages 55-73 years) shared similar views of social media, while GenX (ages 39-54 years) tended to be the least optimistic. Prior studies show that older internet users are less optimistic about social media [26]; however, the common notion that older practitioners are less likely to adapt to emerging technologies may not be true [27]. The discovery that millennials have more optimism regarding social media may not be surprising; however, the shift in demographics is important, as millennials are now the largest proportion of the adult US population [28]. It is likely that millennial and Generation Z (ages 7-23 years) patients will drive an increased need for quality dermatologic information on social media. This underscores the importance of mitigating risks to encourage dermatologists of all generations to engage on social media.

Finally, our survey found that educational and collaborative capabilities were cited among the key advantages of using social media. The rapid dissemination and easy accessibility of new treatments, interesting cases, and continuing medical education through social media highlight this benefit. The perceived benefit of educational opportunities stands in contrast to the lower utilization of social media by academic dermatologists uncovered in our survey. A list of the top influencers in dermatology was recently published [14]; however, less than half (14/30) were faculty at academic institutions, highlighting an opportunity for academic dermatologists to engage on social media.

### Limitations

Our study was limited by a small sample size. Our sample population demonstrates similar demographics to other studies [29]; however, few responses from baby boomers require caution in the interpretation of our generational results. Similarly, bias toward positive social media perceptions may have been introduced by delivering our survey using a social media platform. The APD listserv was utilized to mitigate this bias and increase the practice diversity of our cohort.

Our survey’s completion rate was 77.7% (128/166); however, a response rate could not be calculated since our survey was posted online and was shareable. We mitigated this by tracking clicks on our survey, which produced 166 respondents.

### Conclusion

Our survey identified risks that act as barriers and perceived benefits driving increased social media usage. Views varied significantly among generations, with millennial and baby boomer dermatologists expressing more optimistic outlooks than Generation X. Our research can be used to develop best practices to mitigate risks of privacy violation, litigation, and poor patient care, while promoting education and collaboration can help shape the presence of dermatology on social media.

## Appendix

Multimedia Appendix 1 Web-based survey assessing dermatologists' social media perceptions.

## Abbreviations

APD: Association of Professors in Dermatology
