# Supplementary material for: Risks and Benefits of Using Social Media in Dermatology: Cross-sectional Questionnaire Study
Source: JMIR Dermatol. 2021 Feb 24;4(1):e24737. doi: 10.2196/24737 (PMC10334963; doi:10.2196/24737)

Dermatologists Social Media Perceptions

Welcome to the Survey

Please help share your perceptions of using social media in Dermatology.

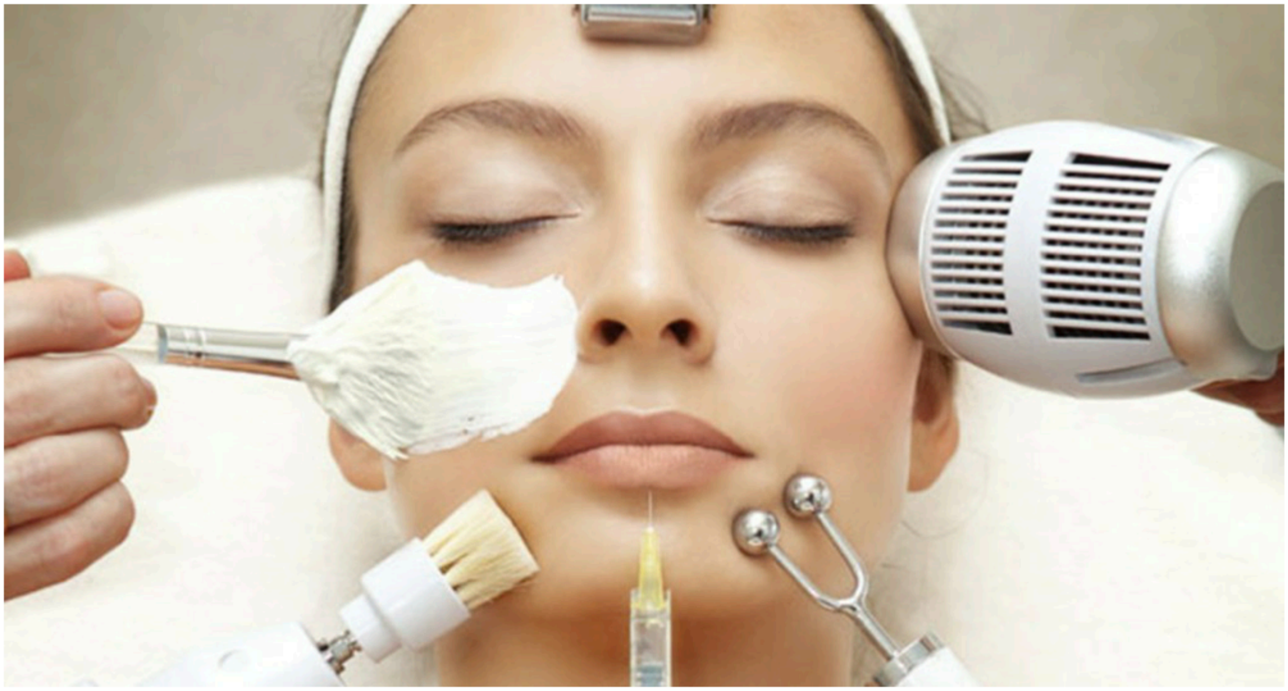

Take the survey!

Dermatologists Social Media Perceptions

Qualifications

* 1. In what country do you currently reside?

- United States
- Other (please specify)

* 2. Please check your Dermatology credentials.

- [ ] Board Certified Dermatologist
- [ ] Medical Resident enrolled in a Dermatology Program
- [ ] Non-board certified, board-eligble
- [ ] Non-board certified
- [ ] Other (please specify)

Dermatologists Social Media Perceptions

Demographics

Tell us about yourself. All information will be kept strictly confidential.

3. What state do you reside in?

4. Gender

- ( ) Female
- ( ) Male
- ( ) Prefer not to answer

5. Age

6. Number of years in clinical practice as a physician

7. You are:

- ◯ An owner of a solo practice
- ◯ An equity owner of a group practice
- ◯ An employee of a group practice, hospital, or health care system
- ◯ An employee of an academic institution
- ◯ Mixed practice/other type of practice (please specify)

8. Completed Education Degrees (check all that apply)

- ☐ MD
- ☐ DO
- ☐ Phd
- ☐ Masters
- ☐ MPH
- ☐ MBA
- ☐ Other (please specify)

Dermatologists Social Media Perceptions

Social Media Usages in Health Care Delivery

9. How many years have you been using social media?

10. On which of the following social networking sites are you *currently* active?
(check all that apply)

- ☐ Facebook
- ☐ Instagram
- ☐ LinkedIn
- ☐ Reddit
- ☐ Snapchat
- ☐ Twitter
- ☐ Whatsapp
- ☐ YouTube
- ☐ Other (please specify)

11. Which of the following social networking services is *most valuable* to you?
(pick one)

- ○ Facebook
- ○ Instagram
- ○ LinkedIn
- ○ Reddit
- ○ Snapchat
- ○ Twitter
- ○ Whatsapp
- ○ YouTube

12. On which type of device do you typically access social media? (check all that apply)

- [ ] Smartphone
- [ ] Tablet
- [ ] Computer
- [ ] Smartwatch
- [ ] Other (please specify)

13. Where do you typically use social media? (check all that apply)

- [ ] Home
- [ ] Work
- [ ] During commute

14. In a typical day, how much time do you spend on social networking for **personal use?**

Minutes

15. In a typical day, about how much time do you spend using social media for **professional use?**

Minutes

Dermatologists Social Media Perceptions

Perceptions

Please share your personal viewpoints of the uses of social media.

Social Media Can:

16. Help in the delivery of healthcare

Strongly Disagree | Neutral | Strongly Agree

17. Improve my professional knowledge

Strongly Disagree | Neutral | Strongly Agree

18. Increase collaboration among physicians

Strongly Disagree | Neutral | Strongly Agree

19. Could damage my professional image

Strongly Disagree | Neutral | Strongly Agree

20. Help recruit new patients

Strongly Disagree | Neutral | Strongly Agree

21. Strengthen the Doctor-Patient relationship

Strongly Disagree | Neutral | Strongly Agree

22. Increase patient education

Strongly Disagree | Neutral | Strongly Agree

23. Increase access to care

Strongly Disagree	Neutral	Strongly Agree

24. Social media is a good tool for public health awareness.

Strongly Disagree	Neutral	Strongly Agree

25. Social media is a good tool for increasing patient compliance

Strongly Disagree	Neutral	Strongly Agree

26. Social media could breach patient privacy

Strongly Disagree	Neutral	Strongly Agree

Dermatologists Social Media Perceptions

Please assess the following risks social media poses for Dermatology:

27. Lack of truthfulness

No Risk	Intermediate Risk	Great Risk

28. Emphasis on superficial values

No Risk	Intermediate Risk	Great Risk

29. Promotion of non-evidence based products

No Risk | Intermediate Risk | Great Risk

30. Unqualified providers substituting proper Dermatological care

No Risk | Intermediate Risk | Great Risk

Dermatologists Social Media Perceptions

Please share your personal experience while using social media.

31. Social Media affects relationships with your **Family:**

Very Negatively | Neutrally | Very Positively

32. Social Media affects relationships with your **Friends**:

Very Negatively | Neutrally | Very Positively

33. Social Media affects relationships with your **Professional Colleagues**:

Very Negatively | Neutrally | Very Positively

34. Social Media affects relationships with your **Patients**:

Very Negatively | Neutrally | Very Positively

35. Are you willing to use/increase the use of the social medial in your professional practice in the future?

- ◯ Yes
- ◯ No
- ◯ Maybe

36. Type 3 words that best describe your perceptions of the use of social media in Dermatology. (separate with commas)

Dermatologists Social Media Perceptions

Completion

Congratulations! You just created science!

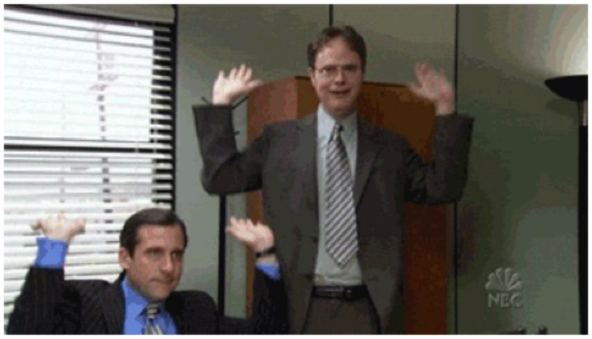

Supplement: Multimedia Appendix 1 [file derma_v4i1e24737_app1.pdf]
